# Supplementary material for: SH3RF3 promotes breast cancer stem-like properties via JNK activation and PTX3 upregulation
Source: Nat Commun. 2020 May 19;11:2487. doi: 10.1038/s41467-020-16051-9 (PMC7237486; doi:10.1038/s41467-020-16051-9)
Supplement: Supplementary file 1 — Supplementary Information [file 41467_2020_16051_MOESM1_ESM.pdf]

Supplementary Information for

SH3RF3 Promotes Breast Cancer Stem-Like Properties via JNK Activation and PTX3 Upregulation

By Zhang et al.

Supplementary Information

Supplementary Information contains 6 Supplementary Figures and 3 Supplementary Tables. The supplementary data files are provided as excel files, separately. And, uncropped images of all Western blots were attached in the source data file.

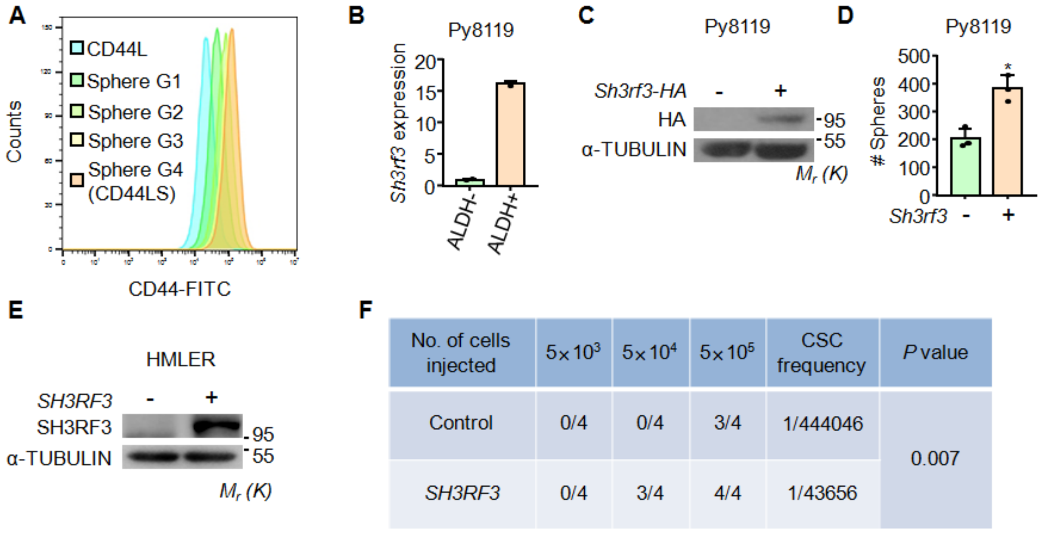

No. of cells injected	5×10³	5×10⁴	5×10⁵	CSC frequency	*P* value
Control	0/4	0/4	3/4	1/444046	0.007
SH3RF3	0/4	3/4	4/4	1/43656	

Supplementary Figure 1. The role of SH3RF3 in BCSC regulation.

(A) Expression of CD44 in CD44L cells in different generations (G1-G4) of consecutive tumorsphere passages. (B) *Sh3rf3* expression in ALDH+ and ALDH- subpopulations of Py8119 (n = 3 qPCR assays). (C) Validation of *Sh3rf3* overexpression in Py8119. (D) Tumorsphere formation of *Sh3rf3*-overexpressing Py8119 (n = 3 culturing experiments). (E) Validation of *SH3RF3* overexpression in HMLER. (F) *In vivo* tumor formation in the mice injected with various numbers of HMLER cells at day 90. The experiments in B and D were repeated three times independently with similar results, and the data of one representative experiment are shown. Data represent mean ± SD; *P < 0.05, **P <0.01. Statistical significance was determined by two-tailed unpaired t-test (b, d) or chi-squared test (f). The experiments in C and E were repeated three times independently with similar results, and the data of one representative experiment are shown. Source data are provided as a Source Data file.

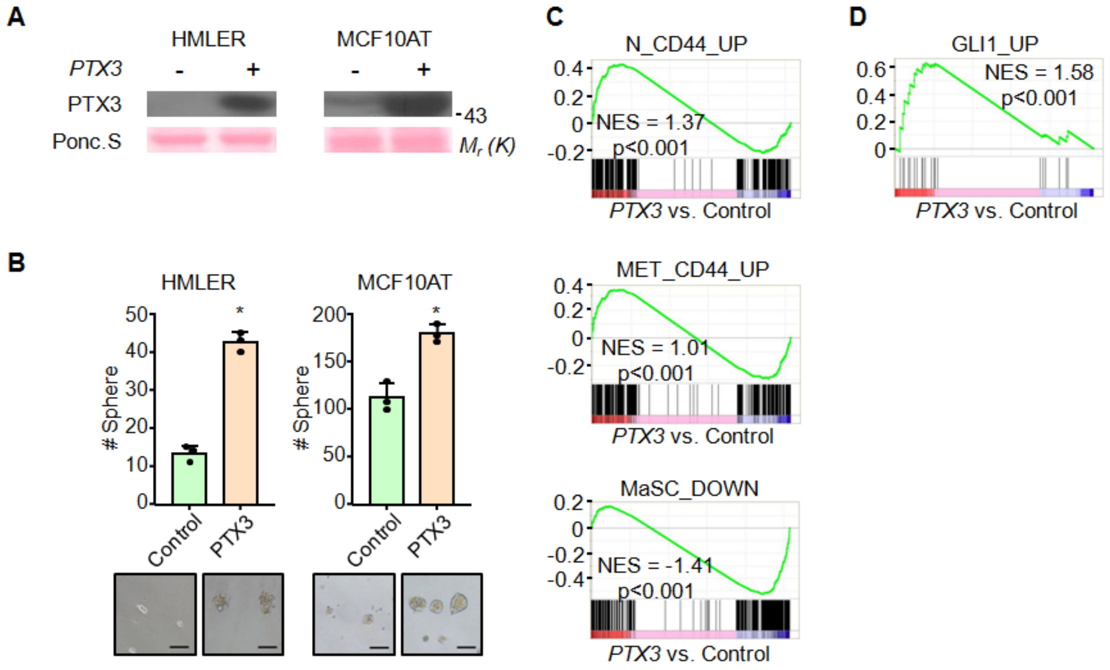

Supplementary Figure 2. The role of PTX3 in BCSC regulation.

(A) Validation of *PTX3* overexpression in HMLER and MCF10AT cells. (B) Quantitation (n = 3 culturing experiments) and representative images of tumorspheres in HMLER and MCF10AT cells after *PTX3* overexpression. (C) GSEA analyses of CSC and MaSC-related gene sets after *PTX3* overexpression. (D) GSEA analyses of Hedgehog-regulated gene sets in *PTX3*-overexpressing versus control cells. The experiments in B were repeated three times independently with similar results, and the data of one representative experiment are shown. Data represent mean ± SD; *$P < 0.05$. Statistical significance was determined by two-tailed unpaired t-test (b). The experiments in A were repeated three times independently with similar results, and the data of one representative experiment are shown. Source data are provided as a Source Data file.

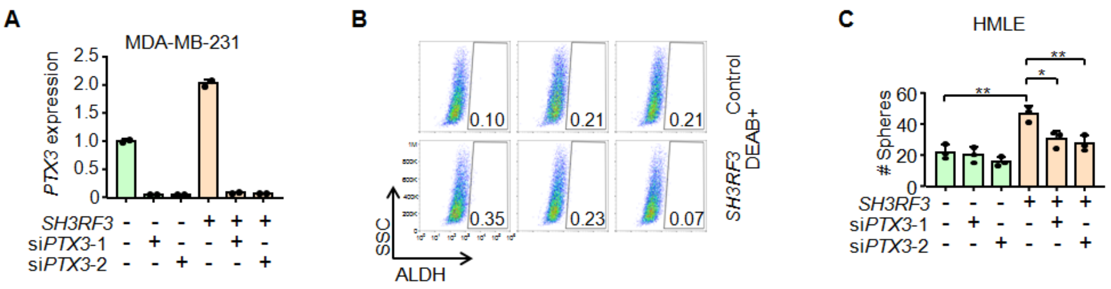

Supplementary Figure 3. PTX3 mediates the function of SH3RF3 in CSC maintenance.

(A) qPCR verification of *PTX3* knockdown in MDA-MB-231 with *SH3RF3* overexpression (n = 3 qPCR assays). (B) DEAB$^+$ negative control for ALDH flow cytometry analyses of MDA-MB-231 with *SH3RF3* overexpression and *PTX3* knockdown. (C) Tumorsphere formation of HMLE with *SH3RF3* overexpression and *PTX3* knockdown (n = 3 culturing experiments). The experiments in A and C were repeated three times independently with similar results, and the data of one representative experiment are shown. Data represent mean ± SD; *P < 0.05, **P <0.01. Statistical significance was determined by two-tailed unpaired t-test (c). Source data are provided as a Source Data file.

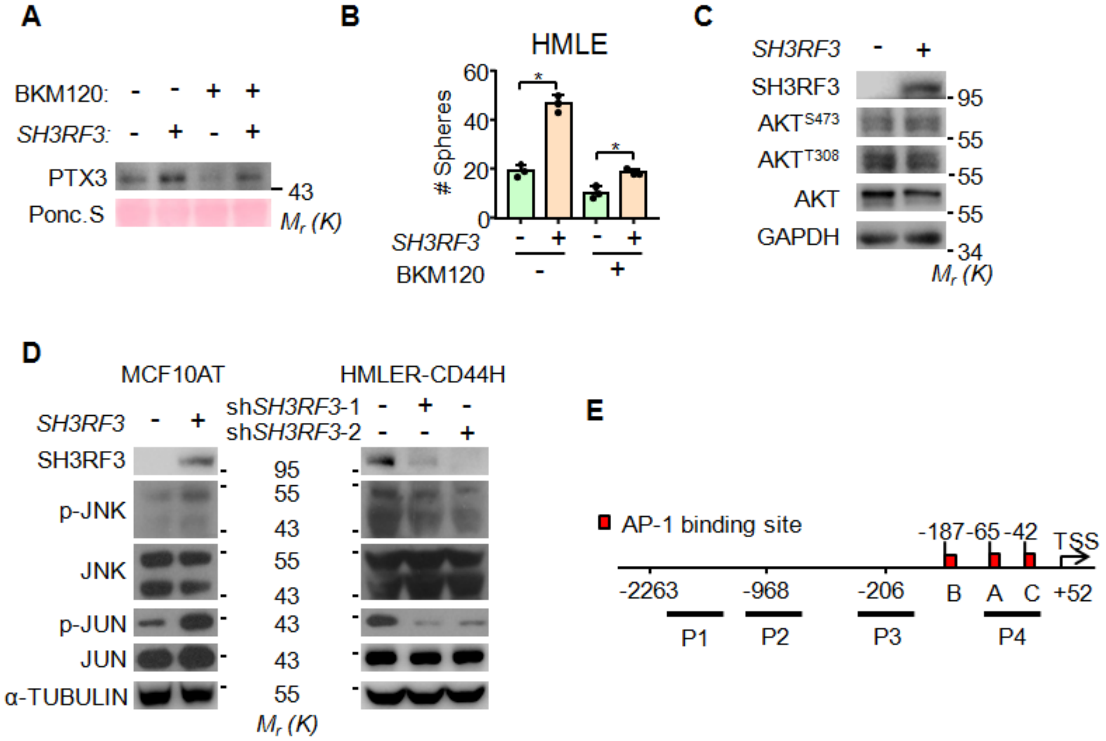

Supplementary Figure 4. *SH3RF3* enhances *PTX3* expression through the JNK-JUN pathway.

(A-B) PTX3 expression (A) and tumorsphere formation (B, n = 3 culturing experiments) of *SH3RF3*-overexpressing and control HMLE cells treated with the PI3K inhibitor BKM120 (1 μM). (C) AKT phosphorylation after *SH3RF3* overexpression in HMLE. (D) JNK and JUN phosphorylation after *SH3RF3* overexpression in MCF10AT and knockdown in HMLER-CD44H. (E) Schematic of the *PTX3* promoter region. P1-P4 indicate the 4 regions analyzed by qPCR after ChIP. A-C indicate the 3 candidate AP-1 binding sites analyzed by mutation followed by luciferase reporter assays. The experiments were repeated three times independently with similar results, and the data of one representative experiment are shown. Data represent mean ± SD; *$P < 0.05$. Statistical significance was determined by two-tailed unpaired t-test (b). Source data are provided as a Source Data file.

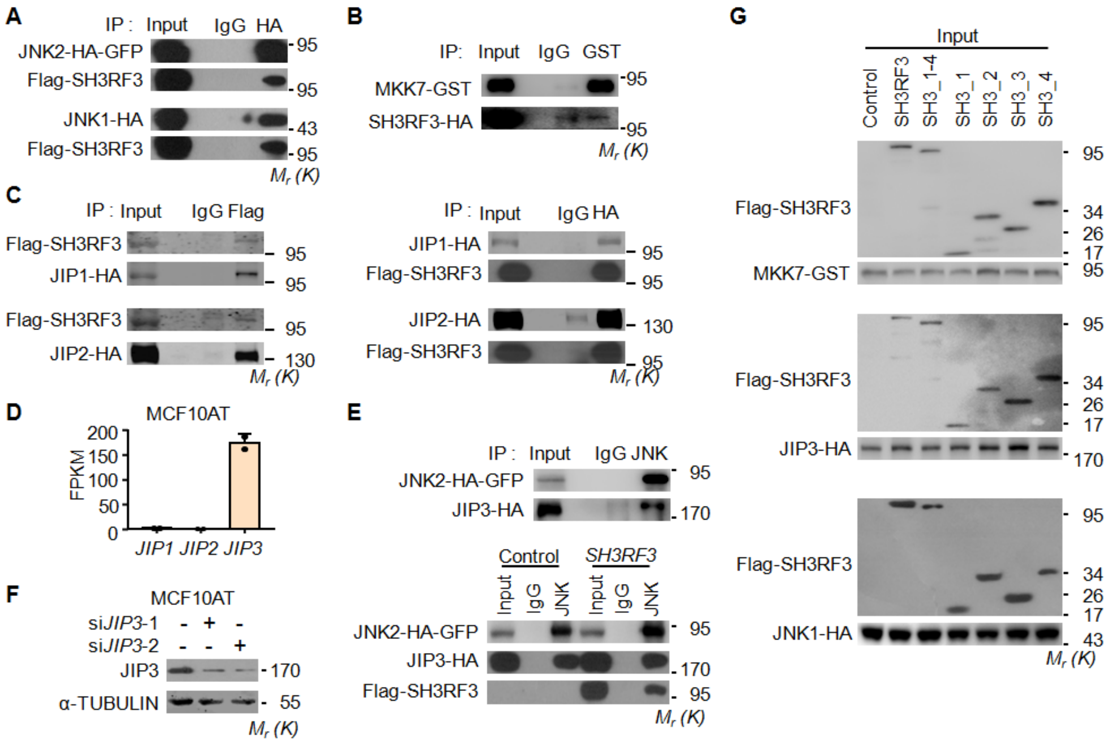

Supplementary Figure 5. SH3RF3 activates JNK-JUN signaling by interacting with MKK and JNK

(A) Co-IP analyses of SH3RF3-JNK1/2 interaction in 293T cells. (B) Co-IP analyses of SH3RF3-MKK7 interaction in 293T cells. (C) Co-IP analyses of SH3RF3-JIP1/2 interaction in 293T cells. (D) Expression of *JIP1/2/3* in MCF10AT cells. (E) Co-IP analyses of JIP3-JNK2 interaction in MCF10AT (top), and with or without *SH3RF3* overexpression (bottom). (F) Validation of *JIP3* knockdown in MCF10AT cells. (G) Input of the Co-IP analyses of SH3RF3 truncations and MKK7, JIP3 and JNK1 in Fig. 7H. The experiments were repeated three times independently with similar results, and the data of one representative experiment are shown. Source data are provided as a Source Data file.

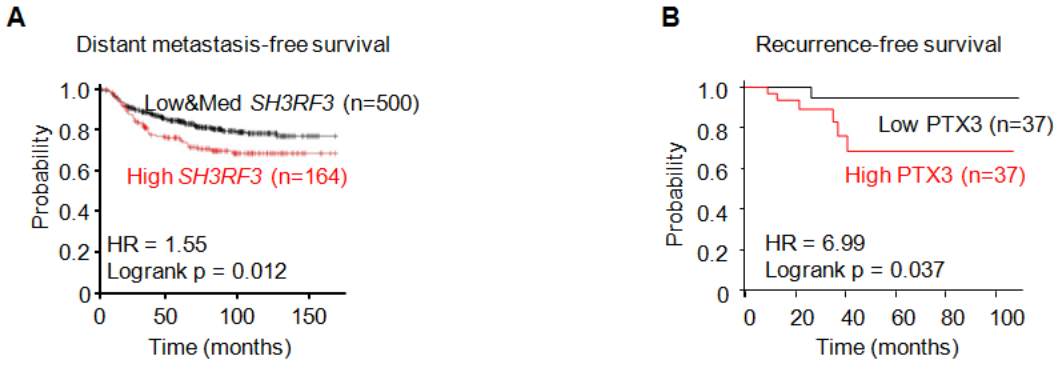

Supplementary Figure 6. Correlation of SH3RF3 and PTX3 expression to breast cancer prognosis.

(A) Distant metastasis-free survival by *SH3RF3* mRNA expression of the Kaplan-Meier Plotter cohort. (B) Recurrence-free survival analysis by PTX3 protein expression of the CPTAC breast cancer cohort. Statistical significance was determined by two-sided log-rank test.

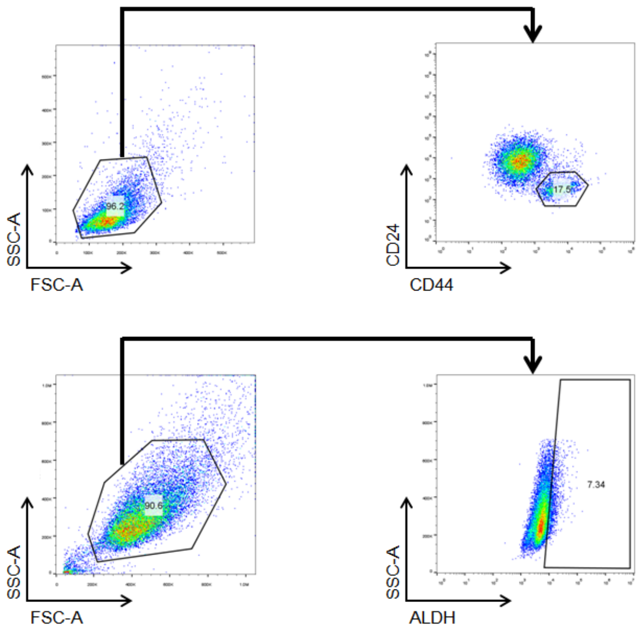

Supplementary Figure 7. Flow cytometric analysis of cancer stem-like cells

Gate strategy of $CD44^{+}CD24^{-}$ (upper) and $ALDH^{+}$ (lower) cancer stem-like cells. $CD44^{+}CD24^{-}$ used in fig 1A, D; 2B; 3B; 4F and supplementary Fig 1A. $ALDH^{+}$ used in fig 1D; 2B; 3B; 5E and supplementary Fig 3B.

.

**Supplementary Table 1. BCSC-associated candidate genes identified by analysis of clinical datasets.**		
Gene symbol	Pearson's r to BCSC ssGSEA scores	logFold (Tumor spheres/ primary tumors)
FBLN5	0.706	2.68
PPAPDC3	0.700	2.60
TMEM119	0.704	2.59
SRPX	0.787	2.52
ACVRL1	0.724	2.51
GPR124	0.763	2.30
LAMB1	0.742	2.19
ITPRIP	0.739	2.17
NNMT	0.717	2.10
ACSL4	0.707	2.07
MRGPRF	0.705	2.07
LRP1	0.786	2.06
NPR2	0.709	2.01
GFPT2	0.754	1.90
TSPAN11	0.705	1.86
ST3GAL2	0.717	1.85
SYDE1	0.713	1.82
EVC2	0.732	1.71
SH3RF3	0.726	1.67
GAS7	0.719	1.60
PDGFRA	0.762	1.52
SNAI2	0.706	1.47
COL6A2	0.718	1.46
CAV1	0.720	1.46
MAF	0.702	1.42
FMNL3	0.731	1.41
MMP2	0.760	1.36
PTRF	0.806	1.34
CARD6	0.719	1.29
DAB2	0.714	1.28
HSPG2	0.755	1.26
SERPINF1	0.751	1.25
GSN	0.698	1.24
TIMP2	0.723	1.20
ANXA1	0.711	1.17
GLI2	0.763	1.14
MRC2	0.714	1.12
RASA3	0.698	1.10
CALD1	0.758	1.09

ANTXR2	0.730	1.08
FAM126A	0.715	1.01
PARD6B	-0.409	-4.31
TPD52	-0.452	-4.01
GINS2	-0.417	-3.44
ESRP1	-0.404	-3.05
MTL5	-0.438	-2.76
STRBP	-0.487	-2.65
GRHL2	-0.429	-2.40
GKAP1	-0.395	-2.28
AP1M2	-0.410	-2.25
MARVELD2	-0.392	-2.17
TMEM106C	-0.434	-2.06
LOC81691	-0.409	-2.05
SLC9A3R1	-0.473	-2.03
C2orf15	-0.407	-2.00
DLG3	-0.418	-1.99
IRX5	-0.397	-1.83
METTL2A	-0.403	-1.73
CYB561	-0.438	-1.61
C17orf75	-0.460	-1.60
C17orf58	-0.486	-1.55
ESRP2	-0.392	-1.54
YEATS4	-0.395	-1.51
C12orf73	-0.515	-1.47
SUDS3	-0.427	-1.46
C6orf211	-0.437	-1.38
POLR2K	-0.418	-1.35
FANCF	-0.400	-1.34
ERI2	-0.411	-1.22
PHKA1	-0.395	-1.20
SNRNP25	-0.436	-1.16
HN1L	-0.404	-1.15
MRPS23	-0.475	-1.07
ARL6IP1	-0.437	-1.01
MEAF6	-0.404	-1.01
TMEM68	-0.425	-1.00

Supplementary Table 2. Tumorsphere culture conditions for different cell lines.

Cell line	Medium	Annexing agents	Cell # seeded	Culture time
MCF10AT	DMEM/F12	1:50 B27, 20ng/mL EGF, 10ng/mL bFGF, 4μg/mL heparin, 5μg/m insulin, 0.5μg/mL Hydrocortizone	5000	2 weeks
MCF10CA1h	DMEM/F12		5000	2 weeks
MDA-MB-231	DMEM	1:50 B27, 20ng/mL EGF, 20ng/mL bFGF, 10μg/mL heparin	5000	2 weeks
HMLE	MEBM	1:50 B27, 20ng/mL EGF, 20ng/mL bFGF, 4μg/mL heparin	10000	1 week
HMLER	MEBM		10000	1 week

Supplementary Table 3. Primers used in the study.	
SH3RF3-F	CGGAATTCATGCTGCTCGGAGCGTCCTGGCTG
SH3RF3-R	CGGGATCCTCTCAGAAGCTCTCGACGAAG
SH3RF3-FLAG-F	CGGAATTCCTGCTCGGAGCGTCCTGGCTG
SH3RF3-HA-R	CGGGATCCTCAAGCGTAGTCTGGGACGTCGTATGGGTAG AAGCTCTCGACGAAGCTG
SH3RF3.Ring-R	CGGGATCCCTACACCAGGATGCGGCACTC
SH3RF3.S1-FLAG-F	CGGAATTCGGCTGCGGCGTGGACGAACTG
SH3RF3.S1-R	CGGGATCCCTATGGCAAGGGCTGGATGCA
SH3RF3.S2-FLAG-F	CGGAATTCCACGCCCCGCCCCAGGGAAAA
SH3RF3.S2-R	CGGGATCCCTAGCCCTGCTCTCCAGACAC
SH3RF3.S3-FLAG-F	CGGAATTCACGCCTCCCAAGGTCCAGCTG
SH3RF3.S3-R	CGGGATCCCTACACCGAGTGGGGCCTGAG
SH3RF3.S4-FLAG-F	CGGAATTCGTGTCCCCGCAGCACAGCCAC
Sh3rf3-nF	CGGGATCCATGCTGCTTGGGGCGTCCTGGCT
Sh3rf3-HAtag-R	CGGAATTCTCAAGCGTAGTCTGGGACGTCGTATGGGTACT TCTGAGCAAGGCATTGAAC
PTX3-F	CGGGATCCATGCATCTCCTTGCGATTCTG
PTX3-R	CGGGATCCTTATGAAACATACTGAGCTCC
MKK4-F	CCGCTCGAGATGGCGGCTCCGAGCCCGA
MKK4-GST-R	CGGGATCCATCGACATACATGGGAGAGCT
MKK7-F	CCGCTCGAGATGGCGGCGTCCTCCCTGGAA
MKK7-GST-R	CGGGATCCCCTGAAGAAGGGCAGGTG
JUN-F	CGGAATTCATGACTGCAAAGATGGAAACG
JUN-HA-R	CGGGATCCCTAAGCGTAGTCTGGGACGTCGTATGGGTAA AATGTTTGCAACTGCTGCGT
JNK1-F	CCGCTCGAGCATGAGCAGAAGCAAGCGTGA
JNK1-HA-R	CGGGATCCCTAAGCGTAGTCTGGGACGTCGTATGGGTAT CTACAGCAGCCCAGAG
JNK2GFP-F	CCGCTCGAGATGAGCGACAGTAAATGTGAC
JNK2GFP-HA-R	AGCGTAGTCTGGGACGTCGTATGGGTATCGACAGCCTTCA AGGGGTC
JIP1-F	CGGAATTCATGGCGGAGCGAGAAAG
JIP1-HA-R	CGGGATCCCTAAGCGTAGTCTGGGACGTCGTATGGGTAC TCCAGGTAGATATCTTCTG
JIP2-F	CGGAATTCATGGCGGATCGCGCGGAGA
JIP2-HA-R	CGGGATCCCTAAGCGTAGTCTGGGACGTCGTATGGGTAC TCCAGGTAGATGTCCTCCGT
JIP3-F	GAAGATCTATGATGGAGATCCAGATG
JIP3-HA-R	GAAGATCTCTAAGCGTAGTCTGGGACGTCGTATGGGTACT CGGGGGTGTAGGACA
PTX3por-(-2263)-F	CCGACGCGTCTGAACGTGAGCCCCGACTA
PTX3por-(+52)-R	GAAGATCTGGCGGGAGGAGACTCTCAAG

PTX3por-(-968)-F	CCGACGCGTACCAAGTTATGAAAAGAAACA
PTX3por-(-206)-F	CCGACGCGTGACCCTCCTCCAATTAATCTG
PTX3por-(-111)-F	CCGACGCGTCCCCACCAAATTCAGGGGAAC
PTX3por-delA-F	GCCACCAGCATTCATCCCCATTCAGGCTTTCCTCAGCATT TATTAAGGAC
PTX3por-delA-R	ATGCTGAGGAAAGCCTGAATGGGGATGAATGCTGGTGGC ACTGCGGTAAC
PTX3por-delB-F	CCGACGCGTCGTAAACCTTTGCGGTTTAAT
PTX3por-delC-F	TCCCCATTCAGGTTATTAAGGACTCTCTGCTCCAGCCTCT
PTX3por-delC-R	AGTCCTTAATAACCTGAATGGGGATGAATGCTGGTGGCAC
q-SH3RF3-F	CGGAGCACCATTTCAACA
q-SH3RF3-R	GGTGACACGGTGGCAGTT
q-Sh3rf3-F	GCCAGATGACAAGAAAAACGA
q-Sh3rf3-R	CTCCAGTTCCAAGGCTTCTG
q-PTX3-F	CATCTCCTTGCGATTCTGTTTTG
q-PTX3-R	CCATTCCGAGTGCTCCTGA
q-FADS2-F	CCTTCAGCTGGGAGGAGATTC
q-FADS2-R	CACGAATTCCAGGTCAGGGT
q-HHIP-F	GCTCTGTCGAAACGGCTACT
q-HHIP-R	TCTGATCAAGAATACCTGCCCT
q-WEE1-F	CCACACAAGACCTTCCGCA
q-WEE1-R	CACTTGAGGAGTCTGTCGCA
q-RRM2-F	TTCTTTGCAGCAAGCGATGG
q-RRM2-R	GCCCAGTCTGCCTTCTTCTT
q-SDC2-F	GAGTCGAGAGCAGAGCTGAC
q-SDC2-R	GCGTCGTGGTTTCCACTTTT
q-SLIT2-F	GGCAGCCCTACTGTGAATG
q-SLIT2-R	CCTTTCCCCTCGACAAGAG
q-CTGF-F	ACCGACTGGAAGACACGTTTG
q-CTGF-R	CCAGGTCAGCTTCGCAAGG
q-DLC1-F	CCGTGCTTGATGTGCAGAAAG
q6-DLC1-R	ACCAGTTGCCCGTAGCCAAT
q-EGFR-F	TATTGATCGGGAGAGCCGGA
q-EGFR-R	TGCGTGAGCTTGTTACTCGT
q-MMP3-F	GGTTCCGCCTGTCTCAAGAT
q-MMP3-R	AGGGATTTGCGCCAAAAGTG
q-CD44-F	GACACCATGGACAAGTTTTGG
q-CD44-R	CGGCAGGTTATATTCAAATCG
q-MGST1-F	TCGGCCTCACCACCAAAATTGA
q-MGST1-R	TTGCCAAATGCTACACAGTCTTC
q-ENPP2-F	TGGGCTGCACTTGTGATGAT
q-ENPP2-R	TCAGGAACGCTGGAAACCTC
q-PTX3por-F1	TTTCGTCCTCCTGAACAATGA

q-PTX3por-R1	GTGGCAAATTAGGCAAAGCTG
q-PTX3por-F2	ATCCTTGCCTCGAAACCTTGT
q-PTX3por-R2	ACCCGGCCTATGACAAACAAC
q-PTX3por-F3	ACAGCTCACAGAAAATGCTGA
q-PTX3por-R3	GGGTCAGAGGGGAAAGAGGAG
q-PTX3por-F4	GCAGTGCCACCAGCATTACTC
q-PTX3por-R4	CGGGAGGAGACTCTCAAGTGA
