# Supplementary material for: SH3RF3 promotes breast cancer stem-like properties via JNK activation and PTX3 upregulation
Source: Nat Commun. 2020 May 19;11:2487. doi: 10.1038/s41467-020-16051-9 (PMC7237486; doi:10.1038/s41467-020-16051-9)
Supplement: Supplementary file 3 — Description of Additional Supplementary Information [file 41467_2020_16051_MOESM3_ESM.pdf]

Description of Additional Supplementary Files

File Name: Supplementary Data 1
Description: Gene differentially expressed in HMLER sublines.

File Name: Supplementary Data 2
Description: Gene sets used for GSEA and ssGSEA analyses.
