# Supplementary material for: SH3RF3 promotes breast cancer stem-like properties via JNK activation and PTX3 upregulation
Source: Nat Commun. 2020 May 19;11:2487. doi: 10.1038/s41467-020-16051-9 (PMC7237486; doi:10.1038/s41467-020-16051-9)
Supplement: Supplementary file 6 — Reporting Summary [file 41467_2020_16051_MOESM6_ESM.pdf]

nature research

Corresponding author(s): Guohong Hu

Last updated by author(s): Mar 23, 2020

Reporting Summary

Nature Research wishes to improve the reproducibility of the work that we publish. This form provides structure for consistency and transparency in reporting. For further information on Nature Research policies, see Authors & Referees and the Editorial Policy Checklist .

Statistics

For all statistical analyses, confirm that the following items are present in the figure legend, table legend, main text, or Methods section.

n/a	Confirmed	
☐	☒	The exact sample size (*n*) for each experimental group/condition, given as a discrete number and unit of measurement
☐	☒	A statement on whether measurements were taken from distinct samples or whether the same sample was measured repeatedly
☐	☒	The statistical test(s) used AND whether they are one- or two-sided<br>*Only common tests should be described solely by name; describe more complex techniques in the Methods section.*
☒	☐	A description of all covariates tested
☐	☒	A description of any assumptions or corrections, such as tests of normality and adjustment for multiple comparisons
☐	☒	A full description of the statistical parameters including central tendency (e.g. means) or other basic estimates (e.g. regression coefficient) AND variation (e.g. standard deviation) or associated estimates of uncertainty (e.g. confidence intervals)
☐	☒	For null hypothesis testing, the test statistic (e.g. *F*, *t*, *r*) with confidence intervals, effect sizes, degrees of freedom and *P* value noted<br>*Give P values as exact values whenever suitable.*
☒	☐	For Bayesian analysis, information on the choice of priors and Markov chain Monte Carlo settings
☒	☐	For hierarchical and complex designs, identification of the appropriate level for tests and full reporting of outcomes
☐	☒	Estimates of effect sizes (e.g. Cohen's *d*, Pearson's *r*), indicating how they were calculated

Our web collection on statistics for biologists contains articles on many of the points above.

Software and code

Policy information about availability of computer code

Data collection	Immunofluorescent staining data were collected by ZEN (blue edition) 2.3 software (Zeiss, Germany).
Data analysis	R version 3.1.2 and GraphPad Prism 7 was used to draw graphs and analyze statistical data. FlowJo_V10 was used to analyze flow cytometry data. Immunofluorescent staining data were analyzed by ZEN software (Zeiss, Germany). Gene set enrichment analysis (GSEA) were analyzed by gsea2-2.2.4.jar. R version 3.1.2. RNA-seq data were analyzed by Skewer v0.2.2, STAR v2.5.1b, Picard2.21.9, RSEM 1.2.29 and EdgeR v3.8.5.

For manuscripts utilizing custom algorithms or software that are central to the research but not yet described in published literature, software must be made available to editors/reviewers. We strongly encourage code deposition in a community repository (e.g. GitHub). See the Nature Research guidelines for submitting code & software for further information.

Data

Policy information about availability of data

All manuscripts must include a data availability statement. This statement should provide the following information, where applicable:

- Accession codes, unique identifiers, or web links for publicly available datasets
- A list of figures that have associated raw data
- A description of any restrictions on data availability

The RNA sequencing data of HMLER CD44L, CD44H, CD44LS, SH3RF3-overexpressing, PTX3-overexpresssing and control cells, related to Fig 1E, 4A, 4H, 4I, 5B, 5C and S1C, were deposited in NODE database (OEP000303, OEP000304 and OEP000305) and GEO database (GSE130577) in Page 28 in manuscript. All the data are released, and everyone can download it for free.

Field-specific reporting

Please select the one below that is the best fit for your research. If you are not sure, read the appropriate sections before making your selection.

☒ Life sciences ☐ Behavioural & social sciences ☐ Ecological, evolutionary & environmental sciences

For a reference copy of the document with all sections, see nature.com/documents/nr-reporting-summary-flat.pdf

Life sciences study design

All studies must disclose on these points even when the disclosure is negative.

Sample size	Sample sizes for each experiment are stated in figure legends. Sample sizes are determined empirically, and similar in size to most existing studies in the same field. For in vitro assays, n=3-4. For in vivo mouse experiments, usually n = 4-10 of mice were used for each group and occasionally 1-2 mice were dropped out for further analyses due to accidental deaths of the animals. No statistical method was used to predetermine sample size.
Data exclusions	Usually no data were excluded, except in the in vivo mouse experiments when 1-2 mice were dropped out for further analyses due to accidental deaths of the animals.
Replication	The in vitro experiments were repeated at least three times, and the in vivo experiment results were a combination of at least two independent repeated experiments.
Randomization	Mice were randomly allocated among groups.
Blinding	This study only involves animal experiments. Investigators were not blinded during mouse grouping for serial dilution tumorigenesis analyses since the animal experiments were performed by the same researchers for data consistency, grouping blinding was impossible. Since the mice were randomly allocated among groups, blinding should not be relevant in these analyses.

Reporting for specific materials, systems and methods

We require information from authors about some types of materials, experimental systems and methods used in many studies. Here, indicate whether each material, system or method listed is relevant to your study. If you are not sure if a list item applies to your research, read the appropriate section before selecting a response.

Materials & experimental systems

n/a	Involved in the study	
☐	☒	Antibodies
☐	☒	Eukaryotic cell lines
☒	☐	Palaeontology
☐	☒	Animals and other organisms
☐	☒	Human research participants
☒	☐	Clinical data

Methods

n/a	Involved in the study	
☒	☐	ChIP-seq
☐	☒	Flow cytometry
☒	☐	MRI-based neuroimaging

Antibodies

Antibodies used	rabbit anti-human SH3RF3 (ThermoFisher, PA560841), rabbit anti-PTX3 (Proteintech, 13797-1-AP), rabbit anti-human JIP3 (Proteintech, 25212-1-AP), rabbit anti-human JNK2 (Proteintech, 51153-1-AP), rabbit anti-human phospho-JUN (Ser73) (Cell Signaling Technology, 9164S), rabbit anti-human JUN (Cell Signaling Technology, 9165P), rabbit anti-human phospho-SAPK/JNK (Thr183/Tyr185) (Cell Signaling Technology, 4668t), APC mouse anti-human CD24 (Biolegend, 311118), FITC mouse anti-human CD44 (BD Pharmingen, 555478), PE mouse anti-human CD44 (BD Pharmingen, 555479), Alexa Fluor 488 donkey anti-rabbit IgG (Invitrogen, A-21206), Alexa Fluor 647 goat anti-rat IgG (Biolegend, 405416), rabbit anti-human GAPDH (Sigma, G9545), mouse anti-human α-TUBULIN (Sigma, T6199), mouse anti-human β-ACTIN (Sigma, A2228), rat anti-human CD44 (Santa Cruz, sc-18849), mouse anti-HA (Sigma, H9658), mouse anti-Flag (Sigma, F1804), rabbit anti-HA (Cell Signaling Technology, 3724S)
Validation	All antibodies were validated according to respective manufacturer's information and citations except SH3RF3 (ThermoFisher, PA560841). We performed overexpression (figure 2A), knockdown (figure 3A) and truncation (data not shown) assays to validate the applicability of SH3RF3 (ThermoFisher, PA560841) in western blot, and the dilution rate of this antibody in western blot is 1:500.

Eukaryotic cell lines

Policy information about cell lines

Cell line source(s)	HeLa was purchased from from the Cell Bank, Chinese Academy of Sciences. MDA-MB-231 and its derivatives were obtained from Dr. Massague (MSKCC), who established the derivatives from the parental cell line MDA-MB-231 obtained from ATCC. MCF10AT and MCF10CA1h were obtained from Dr. Miller (Wayne State University) who established them (American Journal of Pathology 1996;148(1):313-319). HMLE and HMLER cell lines were obtained from Dr. Weinberg (MIT) who established them in 2001 (Genes Dev. 2001;15(1):50-65).
Authentication	Cell lines were authenticated via STR genotyping.
Mycoplasma contamination	All cell lines were tested negative for mycoplasma contamination. No cell lines adopted in this study is listed in the database of commonly
Commonly misidentified lines (See ICLAC register)	No cell lines adopted in this study is listed in the database of commonly misidentified cell lines maintained by ICLAC.

Animals and other organisms

Policy information about studies involving animals; ARRIVE guidelines recommended for reporting animal research

Laboratory animals	Female NSG mice aged 4-8 weeks were used in all animal studies. Keep in the standard SPF grade animal house.
Wild animals	The study did not involve wild animals.
Field-collected samples	The study did not involve filed-collected samples.
Ethics oversight	All animal studies were conducted according to the guidelines for the care and use of laboratory animals and were approved by Institutional Biomedical Research Ethics Committee of Shanghai Institutes for Biological Sciences.

Note that full information on the approval of the study protocol must also be provided in the manuscript.

Human research participants

Policy information about studies involving human research participants

Population characteristics	Tumors tissue samples from 40 female patients, aged at 28-87 and diagnosed with breast cancer and treated in Qilu Hospital of Shandong University were included in the Immunofluorescent staining analysis. Tumors from another three female patients aged at 32, 61 and 77 were used for PDO analyses. Samples were randomly selected, there is no potential self-selection bias or other biases.
Recruitment	Sample from patients treated in the years of 2013-2014 with paraffin embedded tumors available were included for tissue Immunofluorescent staining. Fresh tumors from patients with surgical treatment in April-September, 2017 with sufficient sample available were used for PDX and PDO culturing.
Ethics oversight	Institutional Research Ethics Committee of Qilu Hospital of Shandong University with informed consent from all subjects

Note that full information on the approval of the study protocol must also be provided in the manuscript.

Flow Cytometry

Plots

Confirm that:

- [x] The axis labels state the marker and fluorochrome used (e.g. CD4-FITC).
- [x] The axis scales are clearly visible. Include numbers along axes only for bottom left plot of group (a 'group' is an analysis of identical markers).
- [ ] All plots are contour plots with outliers or pseudocolor plots.
- [x] A numerical value for number of cells or percentage (with statistics) is provided.

Methodology

Sample preparation	Cancer cells harvested from culture during the logarithmic growth period were resuspended in PBS.
Instrument	MoFlo Astrios EQ Flow Cytometer (Beckman) for sorting and Gallios Analyzer (Beckman) for analysis.
Software	Collection: Summit6.3.1 for sorting and Gallios Software for analysis.<br>Data processing: FlowJov10 (Tree Star, USA).

Cell population abundance	Purity of post-sort fractions are measured by flow for CD44+CD24- or CD44-CD24+, and purity is >98%,respectively. ALDH+ or ALDH- fractions are also measured by flow with a purity is >95%, respectively.
Gating strategy	Starting cells were gated on a linear FSC/SSC plot, and population in viability stain with signal intensity lower than 10^3 was selected as live cells for the further analysis which was shown in all flow figures. Positive/negative populations were determined by FMO controls.

☒ Tick this box to confirm that a figure exemplifying the gating strategy is provided in the Supplementary Information.
